# Long-Term Physical and Mechanical Properties and Microstructures of Fly-Ash-Based Geopolymer Composite Incorporating Carbide Slag

**DOI:** 10.3390/ma14216692

**Published:** 2021-11-06

**Authors:** Xianhui Zhao, Haoyu Wang, Linlin Jiang, Lingchao Meng, Boyu Zhou, Jiashuo Zhang

**Affiliations:** 1 School of Civil Engineering, Hebei University of Engineering, Handan 056038, China; zhaoxianhui@hebeu.edu.cn (X.Z.); mrzhangjswy@163.com (J.Z.); 2 Department of Civil Engineering, Tianjin Ren’ai College, Tianjin 301636, China; jianglinlin@tjrac.edu.cn (L.J.); menglingchao@tjrac.edu.cn (L.M.); 3 School of Civil and Transportation Engineering, Hebei University of Technology, Tianjin 300401, China; 201911601009@stu.hebut.edu.cn

**Keywords:** carbide slag, fly-ash-based geopolymer, strength, porosity, shrinkage, microstructure

## Abstract

The long-term property development of fly ash (FA)-based geopolymer (FA–GEO) incorporating industrial solid waste carbide slag (CS) for up to 360 d is still unclear. The objective of this study was to investigate the fresh, physical, and mechanical properties and microstructures of FA–GEO composites with CS and to evaluate the effects of CS when the composites were cured for 360 d. FA–GEO composites with CS were manufactured using FA (as an aluminosilicate precursor), CS (as a calcium additive), NaOH solution (as an alkali activator), and standard sand (as a fine aggregate). The fresh property and long-term physical properties were measured, including fluidity, bulk density, porosity, and drying shrinkage. The flexural and compressive strengths at 60 d and 360 d were tested. Furthermore, the microstructures and gel products were characterized by scanning electron microscopy (SEM) with energy dispersive spectroscopy (EDS). The results show that the additional 20.0% CS reduces the fluidity and increases the conductivity of FA–GEO composites. Bulk densities were decreased, porosities were increased, and drying shrinkages were decreased as the CS content was increased from 0.0% to 20.0% at 360 d. Room temperature is a better curing condition to obtain a higher long-term mechanical strength. The addition of 20.0% CS is more beneficial to the improvement of long-term flexural strength and toughness at room temperature. The gel products in CS–FA–GEO with 20.0% CS are mainly determined as the mixtures of sodium aluminosilicate (N–A–S–H) gel and calcium silicate hydration (C–S–H) gel, besides the surficial pan-alkali. The research results provide an experimental basis for the reuse of CS in various scenarios.

## 1. Introduction

In recent years, geopolymer materials have obtained much focus, interests, and developments, owing to their environmentally friendly materials that act as an alternative to ordinary Portland cement (OPC) materials [1,2,3]. Geopolymer binders or composites with Si–O–Al stable microstructure are usually synthesized by alkali-activated aluminosilicate, which results in producing some superior properties over OPC in its mechanical strength, hardness, chemical resistance, etc. [4,5,6]. Additionally, geopolymer materials tend to be prepared using industrial waste by-products as an aluminosilicate source (fly ash, red mud, blast furnace slag, etc.) rather than natural materials (including metakaolin, etc.) due to the economic and evenly available characteristics [5,7,8,9]. The reusage of available by-products makes a contribution to promoting the goal of carbon peaking and carbon neutrality in China and protects the international and domestic ecological environment.

At present, low-calcium fly ash (active CaO, lower than 10.0% by mass) is regarded as one of the main industrial solid aluminosilicate by-products, which is employed to synthesize geopolymer materials through alkali activation under a NaOH or/and Na_2_SiO_3_ solution [10]. It is emphasized that, when the concentration of the NaOH solution with less than 10.0 mol/L was used to manufacture the low-calcium fly-ash-based geopolymer (FA–GEO) materials at room temperature, it obtained a lower mechanical strength at a 28-day age and a slower strength enhancement at an early age [11,12,13,14]. The final gel products in FA–GEO were tested as the sodium aluminosilicate (N–A–S–H) gels, which are traditionally called ‘geopolymer gels’ by a series of chemical microscale techniques, including SEM–EDS, XRD, and FTIR tests [15,16]. Moreover, the geopolymer gel was simply defined by chemical components, including the elements of Si, Al, O, Na, K, and Ca, along with its microstructures with the 3D network of the Si–O–Al polymeric bond [17,18]. The definition of geopolymer materials was not mentioned through mechanical properties, which resulted in a wider range. Therefore, it is difficult for the FA–GEO material to be directly utilized in practical engineering, owing to some incompletely reacted fly ash and low-cohesive N–A–S–H gel [19].

Particularly, in previous research, the calcium additives (CaCl_2_, CaO, CaSO_4_, etc.) were verified to enhance the physical and mechanical properties of FA–GEO materials when cured at room temperature [10,20,21]. On one hand, room temperature curing is of great significance for saving energy and realizing in situ pouring. On the other hand, those calcium additives make the Na_2_O–Al_2_O_3_–SiO_2_–H_2_O system shift to become a CaO–Na_2_O–Al_2_O_3_–SiO_2_–H_2_O system for FA–GEO materials, which leads to a final shift in microscale gel products and the macroscale strength [22]. As an industrial calcium-containing by-product, soda residue was mixed as a calcium additive with the FA powder activated by an 8.0 mol/L NaOH solution at room temperature [23]. It was reported that the soda residue effectively improved the early mechanical strength. Besides, the blast furnace slag with high calcium was incorporated as a calcium additive into alkali-activated FA–GEO composites, which makes composites obtain a higher mechanical strength [24]. Adding calcium-containing by-products (soda residue or blast furnace slag, etc.) as calcium sources leads to the production of calcium-containing sodium aluminosilicate ((N,C)–A–S–H) gel, or mixtures of C–S–H and N–A–S–H [25]. The essential changes in gel products and microstructures determine the macro physical and mechanical properties. Therefore, it is significant and prospective that the available calcium-containing by-products, such as calcium additives, can be reused to prepare the geopolymer composites applied, such as some bricks and blocks of building materials, when cured at room temperature.

Moreover, carbide slag (CS), a bulk calcium-containing by-product, is mainly composed of calcium hydroxide from the production mode centered on coal-calcium carbide-acetylene-PVC in the PVC manufacturing industry [26]. Under this production mode, 1.5 to 1.9 tons of carbide slag are produced for every one ton of PVC produced [26]. One ton of calcium carbide reacts with water to produce 0.3 tons of acetylene gas (C_2_H_2_) and 1.2 tons of CS wastes [26]. The current reserves of CS in China have reached more than 10.0 million tons, and the bulk CS occupies land resources for a long time [26]. On one hand, its strong alkalinity changes the pH of soils in the storage area and destroy the original soil; on the other hand, its accumulation will also lead to the enrichment of harmful trace elements. Long-term infiltration will cause groundwater pollution and affect the survival of surrounding residents [27]. Moreover, according to the Identification Standard for Hazardous Wastes in China (GB 5085.7-2007) [28], CS belongs to category II industrial solid waste. Therefore, it is imminent for the resource utilization and green treatment of CS.

In addition, CS was mixed with other solid wastes (such as silicate ash and fly ash, etc.) in order to be an alternative to limestone in cement production, not only reducing the energy consumption and carbon emission, but also inhibiting the auto-shrinkage of cement paste [29]. Lu et al. [27] reported that CS and high-silica limestone (SiO_2_, 18% by mass) were used to synthesize belite–barium calcium sulfoaluminate cement at 1380 °C, and the compressive strengths of 3 d, 7 d, and 28 d reached 29.3, 40.5, and 81.1 MPa, respectively. Sun et al. [30] used CS to synthesize new cementitious materials by blending silica fume (SiO_2_, 94% by mass). The specific surface area of the new material is very similar to Portland cement and exhibits a better porosity. Additionally, Rattanashotinunt et al. [31] found that another new gel material was prepared with CS blended with bagasse (SiO_2_, 55% by mass), which possesses a similar compressive strength to that of OPC, and realizes the 70% replacement of cement. Currently, a hot trend is mixing CS with fly ash (SiO_2_ + Al_2_O_3_ > 70% by mass) to prepare new materials [32,33,34]. However, although CS as a calcium additive was used to improve the properties of materials, the physical and mechanical properties and microstructures of FA–GEO composites incorporating CS is unclear at a long period of up to one year. It is essential to evaluate the influence of CS on the long-term properties of FA–GEO composites as new cementitious materials.

The main aim of this study is to explore the long-term physical and mechanical properties and microstructures of composites, incorporating CS, and to evaluate the influence of CS on FA–GEO composites for up to one year. In this work, FA and CS were examined as solid binder materials to synthesize the FA–GEO composites (mortars), incorporating different CS contents by using NaOH solution as an alkali activator. The fresh, physical, and mechanical properties were investigated, including fluidity, conductivity, bulk density, porosity, drying shrinkage, and flexural and compressive strength. SEM and EDS tests were conducted to clarify the microstructures. The results will provide experimental references for the wider green treatment of CS.

## 2. Materials and Methods

### 2.1. Raw Materials

**Figure 1:**
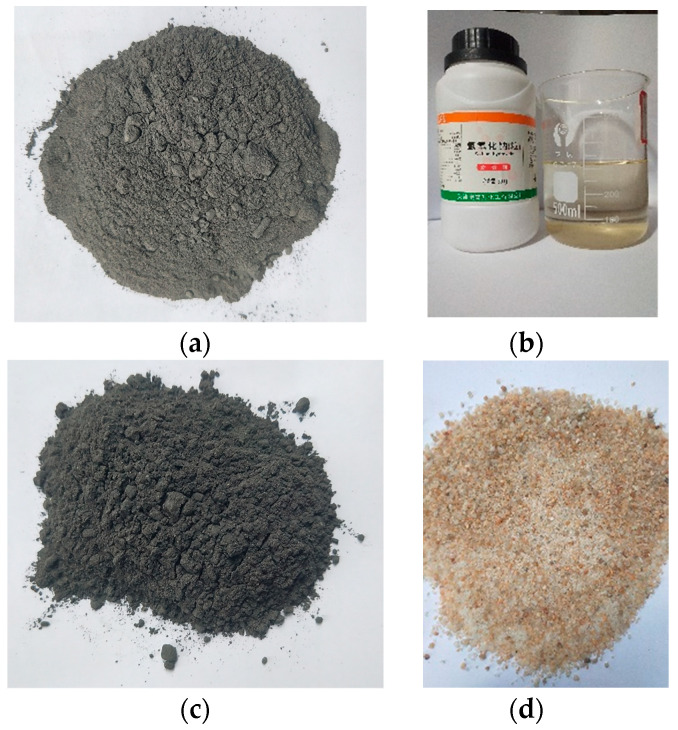
Raw materials for preparing composites: (**a**) fly ash, (**b**) NaOH solution, (**c**) carbide slag, and (**d**) standard sand.

In the present study, carbide-slag-fly-ash-based geopolymer (CS–FA–GEO) composite mortars were synthesized using powder FA as aluminosilicate precursor, powder CS as calcium additive, NaOH solution as alkali-activator, and standard sand as fine aggregate. The used raw materials are shown in Figure 1. The chemical compositions of solid powder FA and CS are present in Table 1, and their basic physical indexes are shown in Table 2.

#### 2.1.1. Solid Powders

As shown in Table 1, the FA is supported by a power plant (Nanning, Jiangxi Province, China). The FA (SiO_2_ + Al_2_O_3_, 76.30% by mass) includes 5.42% CaO, which is classified as low-calcium fly ash (Figure 1a). The CS is derived from a chemical industrial C_2_H_2_ plant (Qinghai of China), which contains 63.13% CaO (Figure 1b). For the physical indexes of FA, the specific gravity reaches 2.45, the specific surface area is 500 m^2^/kg, the amount passing #325 sieve reaches 73%, and the pH measures 5.93 at 100% of water content. Additionally, the physical indexes of CS present the difference in that of FA. For CS, the specific gravity reaches 1.80, the specific surface area is 420 m^2^/kg, the amount passing #325 sieve reaches 40%, and the pH value is 10.55 at 100% of water content, as shown in Table 2. Both the powder FA and powder CS were dried at 40 °C in order to be used.

#### 2.1.2. Alkali-Activated Solution

The used alkaline activator solution (8 mol/L NaOH) was prepared through mixing 3.20 kg of NaOH pellets with 10.00 L of water (25.10% NaOH and 74.90% H_2_O by mass in the solution), as shown in Figure 1c. The NaOH pellets (analytical grade, and higher than 98% purity) were derived from Kemiou Company in Tianjin of China. The experimental tap water was local from Tianjin city of China (pH of water measures 7.15 at room temperature). In the previous study, the 8~14 mol/L NaOH solution was used to achieve the alkali activation. Here, the used 8 mol/L NaOH solution was confirmed as activators regarding economic benefits and the application of bricks in construction materials [15,35]. In this paper, the 8 mol/L NaOH solution was cooled to 25 °C in order to be used before preparation.

#### 2.1.3. Fine Aggregate

Standard sand was utilized as fine aggregate in composite (Figure 1d). The loss on ignition (LOI) of standard sand is less than 0.40%, the mud content (including soluble salts) is less than 0.20%, and the SiO_2_ content is higher than 98%. The standard sand meets the standard of GB/T 17671-1999 (ISO 679:1989, EN 196-1) [36]. The grain size (0.08~2.00 mm), fineness modulus (2.6), density (1500 kg/m^3^), and water absorption (0.5%) of fine aggregate are obtained.

### 2.2. Preparation of Samples

CS–FA–GEO mortar samples were prepared for a total of eleven groups of samples with different CS contents and different pre-curing temperatures of early 2 h. As shown in Table 3, six groups of samples were prepared by incorporating different CS contents (0.0%, 4.4%, 8.8%, 13.2%, 17.6%, and 20.0%) to analyze the effect of CS on physical and mechanical properties under room temperature of 25 ± 2 °C and relative humidity of 50 ± 5% (labeled as T25). Furthermore, to investigate the pre-curing temperature for nearly 2 h as the recommended curing method, five other groups of samples (with identical CS content of 20.0%) were cured under different pre-curing temperatures: 40 ± 2 °C (T40), 50 ± 2 °C (T50), 60 ± 2 °C (T60), 70 ± 2 °C (T70), and 80 ± 2 °C (T80). The 25 ± 2 °C (T25) was regarded as control group. Here, liquid/solid (L/S) ratios and cement/sand (C/S) ratios are shown in Table 3 and Table 4. During pre-treatment, the samples were wrapped in plastic film to avoid moisture loss.

The preparation methods by casting were presented as follows: the solid powders (CS and FA) were first blended for 2 min to become mixture, and then 8 mol/L NaOH solution was blended with the mixture for an additional 3 min according to L/S ratio in Table 3. Furthermore, the standard sand was added by C/S ratio and stirred for 3 min to obtain the fresh mixture. After that, the fresh mixtures were cast into the steel molds (mold size: 40 mm × 40 mm × 160 mm) by two layers, in accordance with the Chinese standard GB/T 17671-1999 (ISO) [36]. Here, each layer of each mixture was vibrated for 2 min. The mold of 40 mm × 40 mm × 160 mm was selected in this paper to compare with the experimental results in the previous study [17,23,37]. All of the samples were cured for 60 d and 360 d under the designed curing methods (in Table 3). The cured samples were demolded at 60 d, owing to the slow hardening at room temperature [10,17,23]. After pre-curing, the identical T25 continued to cure until required age.

In particular, for drying shrinkage samples, the mold of 25 mm × 25 mm × 280 mm was used to measure the shrinkage results of up to 360 d when the samples continued to be cured at room temperature. C/S ratio was set as 1:2, and L/S ratio was set as 0.73. The blending, stirring, and casting in Table 4 were identical to that in Table 3.

### 2.3. Experimental Methods

#### 2.3.1. Determination of Fluidity

**Figure 2:**
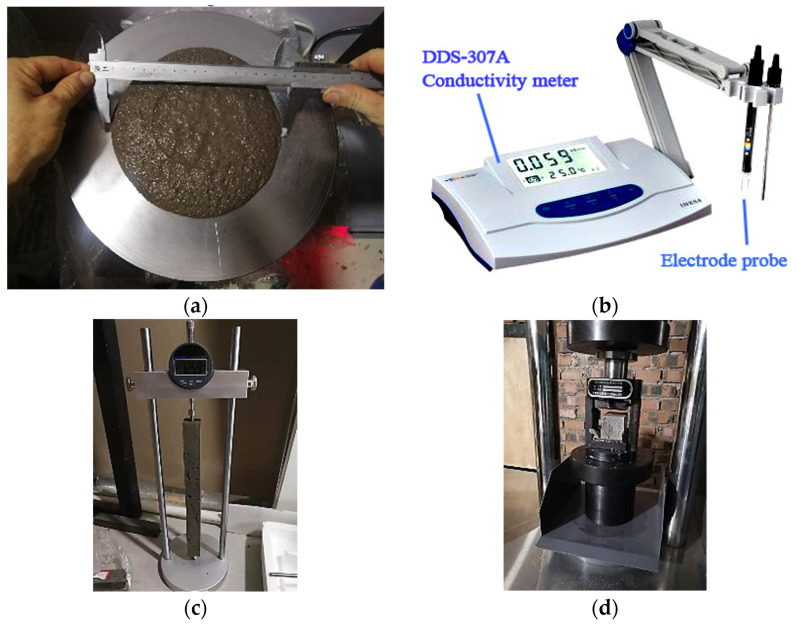
The experiments of (**a**) fluidity, (**b**) conductivity, (**c**) drying shrinkage, and (**d**) mechanical strength.

The fluidity was measured by fluidity tester (jumping table) of the NLD-3 cement mortar in accordance with the Chinese standard of GB/T 2419-2005 [38]. Firstly, the fresh mixtures were cast into a truncated cone mold (60 mm in height, 70 mm in upper inner diameter and 100 mm in bottom inner diameter), as shown in Figure 2a. After removing excessive mixtures and lifting mold vertically, the jumping table experiment begins (with the frequency of 25 times in 25 s by a controller). Afterward, a caliper was used to measure two flow diameters in vertical directions, and the fluidity result was taken from the average. The fluidity tests were finished during ten minutes from the addition of water.

#### 2.3.2. Determination of Conductivity and Total Dissolved Solid (TDS)

To analyze the dissolved ions or cations in the fresh mixtures, a DDS-307A conductivity meter (accuracy 0.01 mS/cm, maximum 100 mS/cm) with an electrode probe (constant 10 cm^−1^) was used to measure the conductivity of fresh mixture. The conductivity was obtained after stirring six minutes and before testing the fluidity. The electrode probe was inserted into the fresh mixture to a depth of two centimeters, and five points were chosen from center and surrounding of probe. This can be seen in Figure 2b. The total dissolved solids (TDS) were also obtained by the relationship of 1 mg/L (TDS) = 2 μS/cm to analyze the dissolved substances in fresh mixtures. The final result was derived from the average of five conductivities due to the heterogeneity.

#### 2.3.3. Determination of Bulk Density and Porosity

The bulk densities of samples cured for 60 d and 360 d were, respectively, tested by drainage method, which is measured by the ratio of mass to volume, and also obtained from the average of six identical samples according to the Chinese standard (GB/T 2542-2012) [39]. Meanwhile, the porosities were tested with ASTM C642-13 in 2013, and the tested method was used as Equation (1):

*POR* = (*m* − *m*_1_)/(*m* − *m*_2_) (1)

Here, *POR*—the porosity (%), *m*—the weight in air of saturated sample (g), *m*_1_—the dry weight of sample after dried for 24 h at 100 °C in the oven (g), and *m*_2_—the weight of sample in water (g) [40]. The porosity results were also obtained from the average of six samples. Moreover, the square areas (40 mm × 40 mm) at the middle positions of side surfaces were uniformly taken out, and the pore distributions were observed by naked eye, as mentioned in the literature [23], in order to analyze the correlation with tested porosity.

#### 2.3.4. Determination of Long-Term Drying Shrinkage

For the drying shrinkage, the mold size of 25 mm × 25 mm × 280 mm was used according to the standard JC/T 603-2004 [41] in China. After being cast into mold, the samples were cured under T25 for 7 d to demold, at which point they were measured as the initial lengths of samples, and, then, they continued to cure under T25 without plastic film. The shrinkages of FC0T25, FC8.8T25, and FC20T25 were recorded for up to 360 d using ratio length meter (BC156-300, Lisheng Instrument Company, Cangzhou of Hebei, China) with a precision of 0.001 mm. This is shown in Figure 2c.

#### 2.3.5. Determination of Long-Term Mechanical Strengths

The flexural and compressive strengths at 60 d, 180 d, and 360 d were tested utilizing a servo-control testing machine (YAW-S, Sansi Zongheng, Shenzhen, China) with a loading capacity of 300 kN, according to GB/T 17671-1999 (ISO). This can be seen in Figure 2d. The loading rates were set as 2400 N/s and 50 N/s for compressive and flexural tests, respectively. The compressive strength results were obtained from the average of six identical samples.

#### 2.3.6. Characterization of Microstructure and Gel Product

The microstructures and gel products of FA, CS, and FC0T25 at 360 d, and FC20T25 at 360 d, were, respectively, detected through SEM and EDS tests. A Quanta FEG450 scanning electron microscope was employed together with an energy dispersive spectroscopy (Hillsboro, OR, USA). The detection method was as follows: the area-scanning technique was used to determine the position of product gels in binders, and then the EDS spectra of representative points were obtained to analyze the gel products [17,19,42]. The samples were used for detection with gold spray pretreatment.

## 3. Results and Discussion

### 3.1. The Fresh Properties of CS–FA–GEO Mixtures

**Figure 3:**
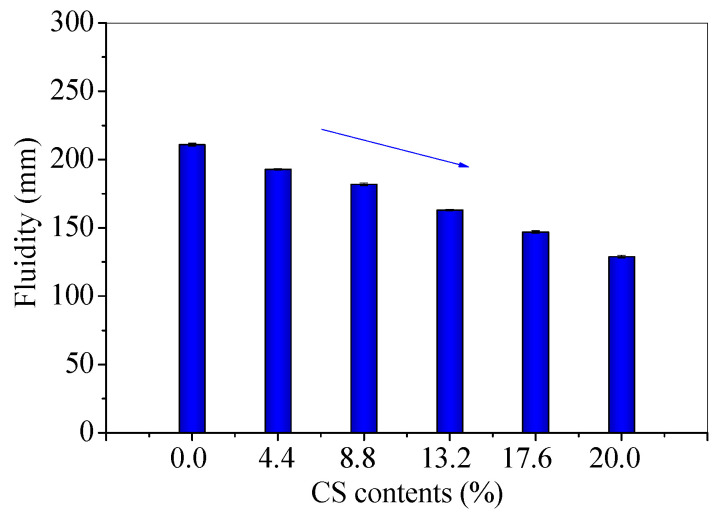
The fluidities of fresh CS–FA–GEO mixtures with different CS contents.

**Figure 4:**
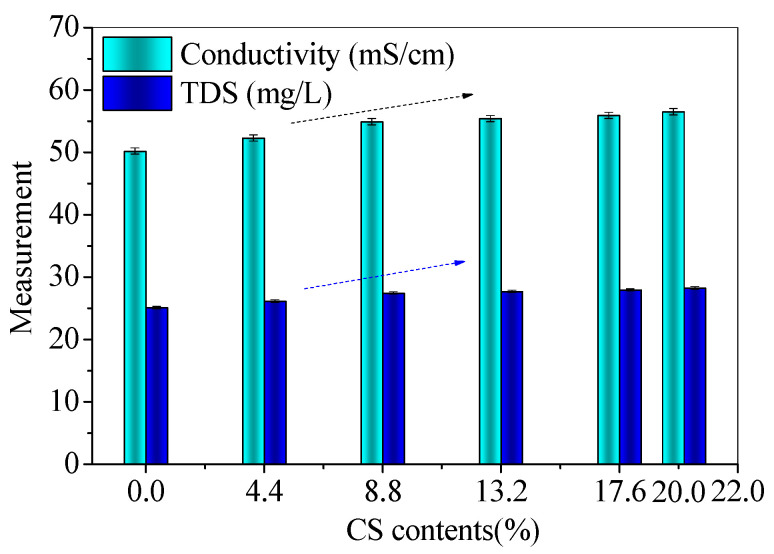
The conductivity and TDS of fresh CS–FA–GEO mixtures with different CS contents.

The fluidity, conductivity, and total dissolved solids (TDS) were measured for fresh mixtures to investigate the influence of CS contents. The fluidity results are shown in Figure 3, and the conductivity and TDS results are present in Figure 4.

With the increase in CS content, the fluidities decrease. The fluidity (129 mm) of FC20T25 reduces by 38.86% than that (211 mm) of FC0T25, which is shown in Figure 3. This may have resulted from the high water absorption of CS, which results from the chemical and physical properties of CS [23]. In addition, the conductivity and TDS increase with the increase in CS content. The conductivity (56.5 mS/cm) of FC20T25 is 12.55% higher than that (50.2 mS/cm) of FC0T25, and the TDS (28.25 mg/L) of FC20T25 is higher than that (25.10 mg/L) of FC0T25, as shown in Figure 4. The increase in conductivity and TDS indicates that there are more conductive dissolved substances in the mixtures. This is because a small amount of free calcium components from CS are dissolved under an alkaline environment, providing a higher ionic environment for further reaction, besides water absorption. This increases the conductivity and reaction probability of anions and cations in fresh mixtures, which accelerates the initial cementation of solid particles. Therefore, the conductivity and TDS increases with an increasing CS ratio, which results from the concentrations of dissolved anions and cations.

As reported in the previous study, the industrial waste soda residue (containing various inorganic calcium salts) was used as a calcium additive to improve the fresh properties of the FA–GEO mortar due to more dissolved Ca^2+^ and the high water absorption of the soda residue [17]. The experiments revealed that the fluidity of the FA–GEO mortar with 20.0% soda residue was 20.1% lower than that without the soda residue. In this research, CS has a similar effect to the soda residue on decreasing the fluidity and conductivity of the FA–GEO samples. Therefore, the addition of 20.0% CS as a calcium additive reduces the fluidity and increases the conductivity of the FA–GEO composites.

### 3.2. The Long-Term Bulk Density of CS–FA–GEO Composites

**Figure 5:**
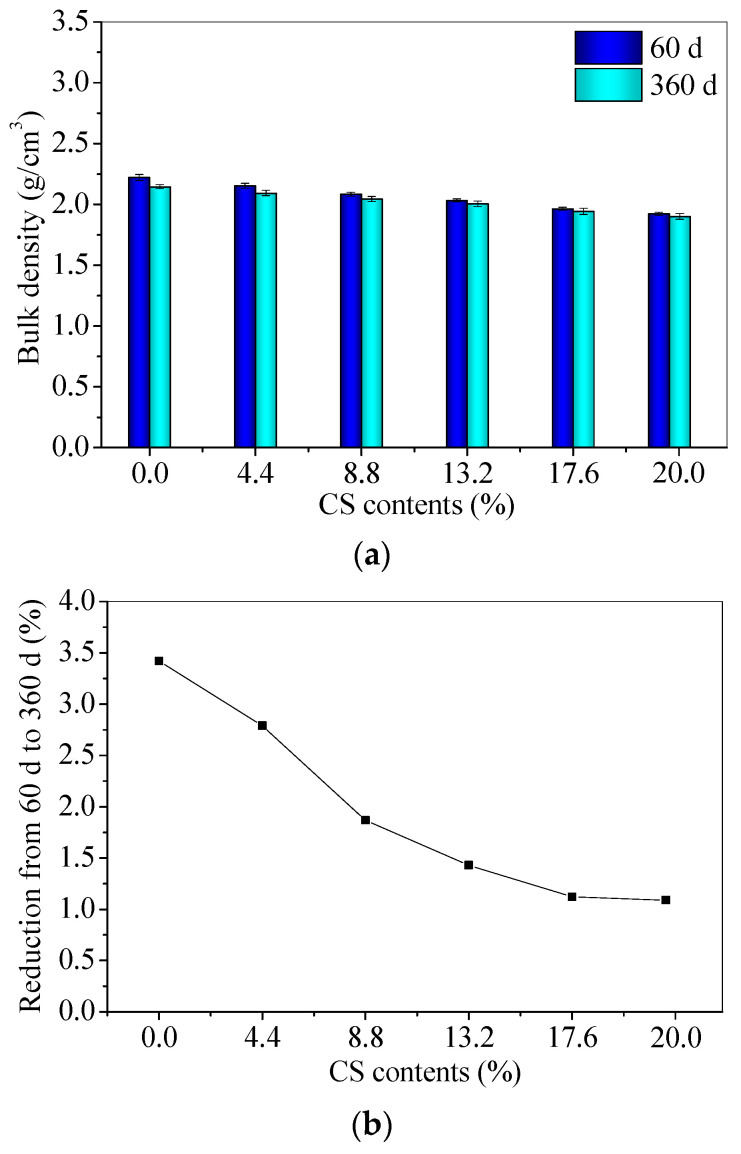
(**a**) Bulk densities and (**b**) reduction percentage of CS–FA–GEO composites with different CS contents.

Figure 5 shows the bulk densities of CS–FA–GEO samples. The 60 d bulk densities of samples decrease as the CS contents increase. The bulk density of FC20T25 is 13.46% lower than that of FC0T25. The decreasing bulk densities of the samples may be mainly attributed to the lower specific gravity of CS (than that of FA). The bulk densities of 360 d are lower than those of 60 d, owing to the loss of water. The decrements from 60 d to 360 d decrease as the CS contents increase, which are in the range of 1.09%~3.42%. The previous research reported that the soda residue was utilized as a calcium additive to prepare the FA–GEO mortar. The bulk density of 150 d obtains 2.096 g/cm^3^ for FA–GEO with a 20.0% soda residue, and the bulk density of mortar with a 20.0% soda residue is slightly lower than that (2.145 g/cm^3^) of mortar without soda residue [17]. In this paper, CS possesses a similar effect to the soda residue on reducing the bulk density. In summary, the addition of 20.0% CS reduces the bulk density of CS–FA–GEO composites.

### 3.3. The Surface Characteristic and Porosity of CS–FA–GEO Composites

**Figure 6:**
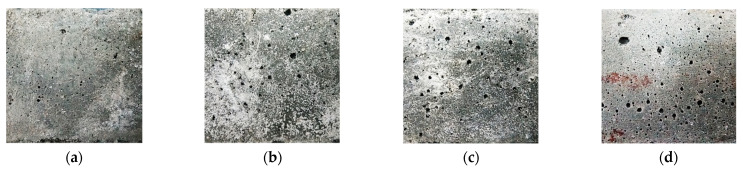
Surficial characteristics of CS–FA–GEO samples: (**a**) FC0T25, (**b**) FC8.8T25, (**c**) FC20T25, and (**d**) FC20T80.

**Figure 7:**
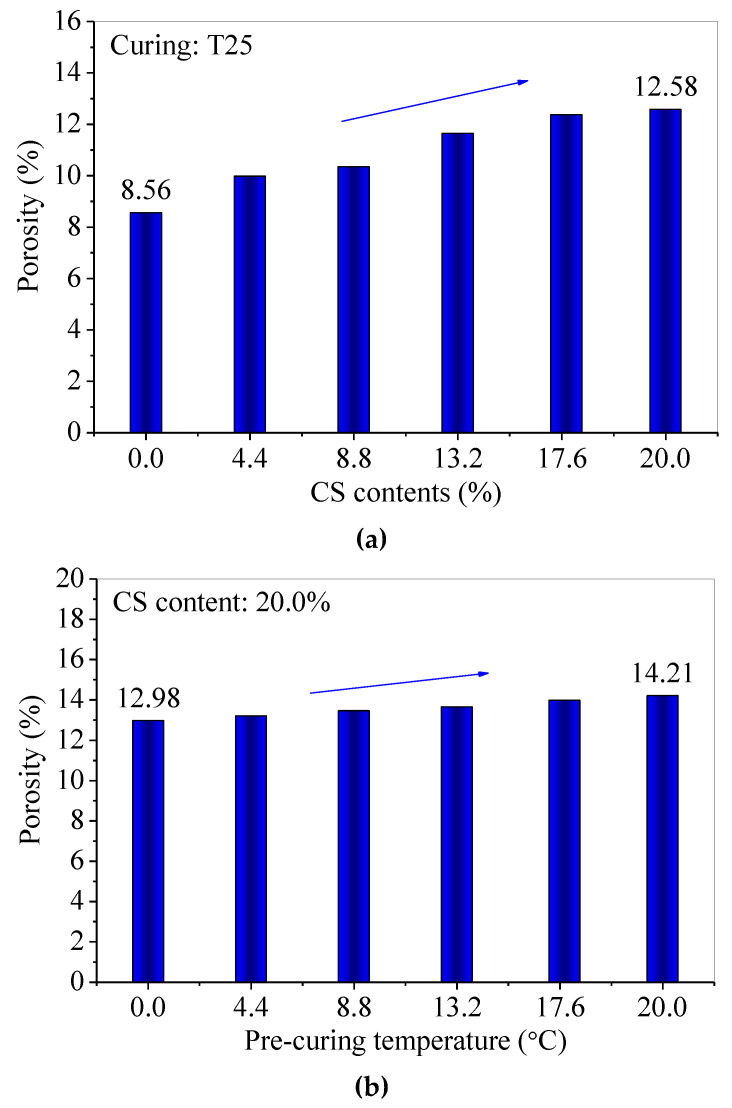
The porosities of CS–FA–GEO samples at 60 d: (**a**) with different CS contents, and (**b**) under different pre-curing temperatures.

Under T25 for 60 d, the surficial pan-alkalis of samples gradually disappear until they are not obviously observed by the naked eye as the CS contents increase from 0.0% to 20.0%; however, the pan-alkali on the surface of FC20T25 (Figure 6) remains. In particular, the pre-curing of 80 °C for nearly 2 h makes the sample FC20T80 produce little pan-alkali at the curing age of 60 d. Moreover, there are many round pores distributed on the surfaces of samples in varying degrees. The round pores distinctly increase with the increase in CS content. The surficial pores of FC20T25 are higher than those of FC0T25. In addition, the measured porosities of samples with different CS contents and different pre-curing temperatures are shown in Figure 7. It can be seen that the porosities of samples increase with the increase in CS content; however, the porosities of samples also increase with the pre-curing temperatures from 25 °C to 80 °C for 2 h.

A previous study reported that a different dosage of soda residue was added into the FA–GEO mortar to investigate the change in porosity [23]. The addition of 20.0% soda residue made the porosity increase from 6.23% to 14.36% compared to that without soda residue, which led to a porosity increment of 1.3 times. However, the addition of 20.0% CS displays the same role in porosity compared to the addition of 20.0% soda residue under T25 for 60 d. Here, the methods of pouring and vibration are identical during the preparation for the same CS content, and, thus, may be because exposure to heat (nearly 2 h pre-curing of higher temperature) contributes to a higher chemical reaction rate and higher pore generation rate, owing to the addition of CS [23]. The increasing CS is added into the fresh mixture, leading to a higher reaction heat. In addition, the high chemical viscosity of fly-ash-based geopolymer forms early, and, also, the reaction heat cannot be released in time, which leads to more porosities [23]. Therefore, the room temperature (25 ± 2 °C, labeled as T25) is recommended to obtain a lower porosity of the CS–FA–GEO composite with 20.0% CS.

### 3.4. The Long-Term Drying Shrinkage of CS–FA–GEO Composites

**Figure 8:**
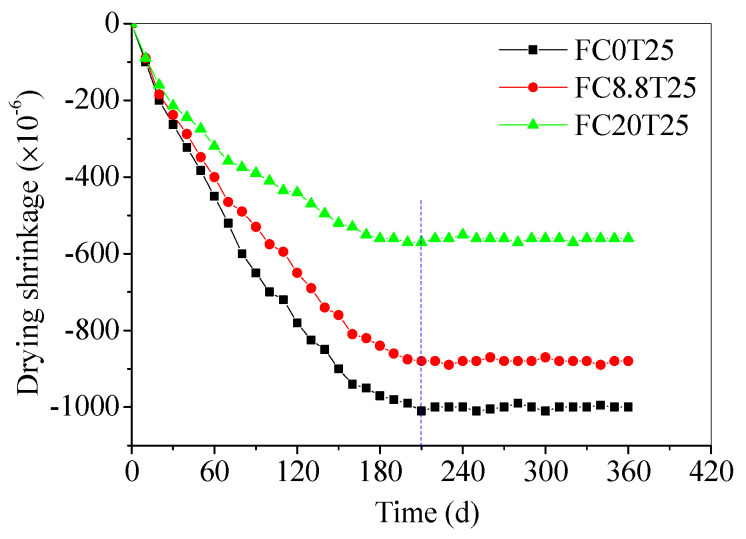
Evolution of drying shrinkage of CS–FA–GEO composites for FC0T25, FC8.8T25, and FC20T25 of up to 360 d.

The long-term drying shrinkage values of samples cured for up to 360 d are presented in Figure 8 to explore the effect of CS in the composites. The FC0T25, FC8.8T25, and FC20T25 were chosen to compare drying shrinkage values at different CS contents. It can be seen that the drying shrinkage values of three samples increase with the curing time, and tend to be stable until 210 d. The shrinkage values reach −560 × 10^−6^ for FC20T25, −880 × 10^−6^ for FC8.8T25, and −1000 × 10^−6^ for FC0T25, respectively. This indicates that the lower drying shrinkage of the FA–GEO composite is obtained with an increase in CS content, which displays that the drying shrinkage properties are improved by additional CS.

In the previous study, Shang, et al. [43] investigated that the 3 d, 7 d, 28 d, and 60 d drying shrinkages of FA–GEO mortars were enhanced by the addition of 20.0% of granulated ground blast-furnace slag (GGBS) as a calcium modifier. The evolution of the drying shrinkage displayed that the addition of 20.0% GGBS obtained the higher drying shrinkage results of the FA–GEO mortars, which is distinctly different from the feature of CS. GGBS was alkali-activated by the activator to shrink due to the characteristics of GGBS being similar to FA. Thus, the larger drying shrinkage was obtained by adding GGBS, including the main compositions of Al_2_O_3_, SiO_2_, and CaO. However, the additional 20.0% CS improves the drying shrinkage property of the FA–GEO mortar, owing to the main composition Ca(OH)_2_ or CaO, which is similar to the feature of high-calcium fly ash. In addition, the differences in the reactivity of the FA, aggregate-to-binder, and water-to-binder between mortars create the distinct drying shrinkage result. In summary, the addition of 20.0% CS has a positive impact on the drying shrinkage of the FA–GEO mortar in construction materials.

### 3.5. The Long-Term Flexural and Compressive Strengths of CS–FA–GEO Composites

**Figure 9:**
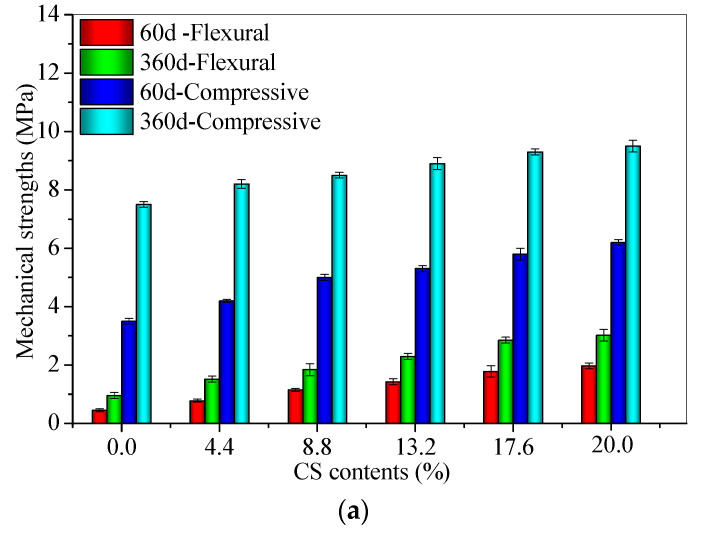

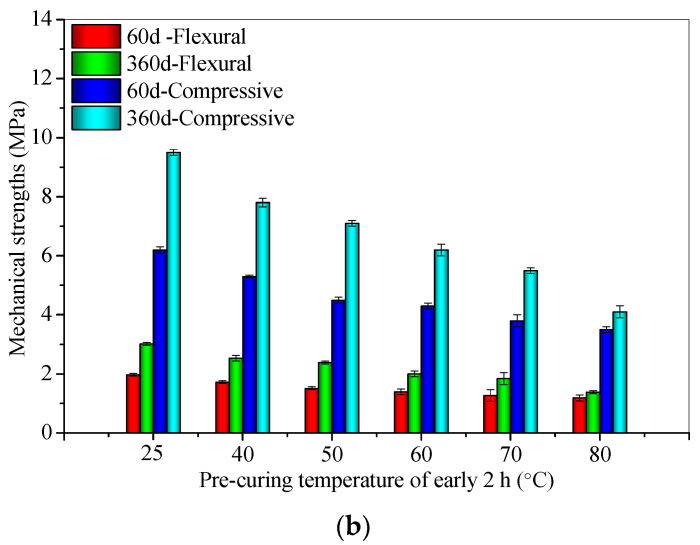
The flexural and compressive strengths of 60 d and 360 d for CS–FA–GEO composites: (**a**) with different CS contents, and (**b**) at different pre-curing temperatures.

**Figure 10:**
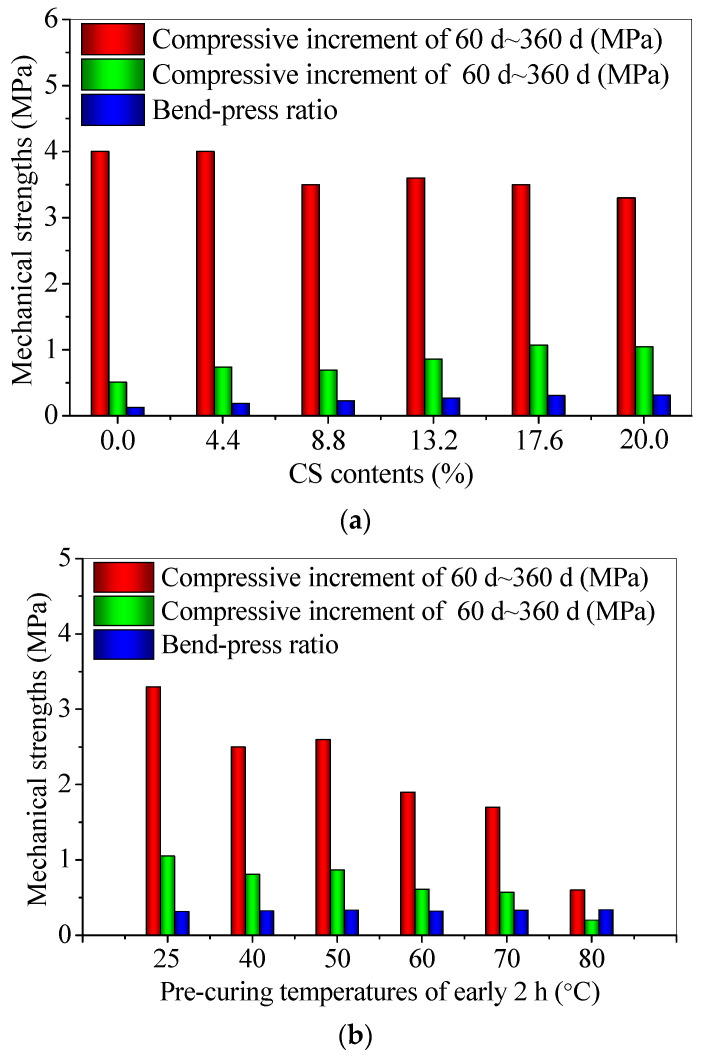
The bend-press ratios and the increments in flexural and compressive strengths of 60 d~360 d for CS–FA–GEO composites: (**a**) with different CS contents, and (**b**) at different pre-curing temperatures.

The measured results of the flexural and compressive strengths of CS–FA–GEO mortars are presented in Figure 9 and Figure 10.

Under T25, with the increase in CS content from 0.0% to 20.0%, the flexural and compressive strengths at 60 d and 360 d increase (Figure 9a). The 60 d flexural strength of FC20T25 reaches 2.0 MPa, which is three times higher than that of FC0T25, whereas the 60 d compressive strength of FC20T25 reaches 6.2 MPa, which is 7.7 times higher than that of FC0T25. Moreover, the 360 d flexural strength of FC20T25 reaches 3.0 MPa, which is two times higher than that of FC0T25, whereas the 360 d compressive strength of FC20T25 reaches 6.2 MPa, which is 26.7% higher than that of FC0T25. A previous study reported that the 20.0% soda residue as a calcium additive was added into FA–GEO composites [23], which performed an enhancement of 60 d flexural strengths from 1.6 MPa to 3.0 MPa, and improved the 60 d compressive strengths from 6.0 MPa to 14.5 MPa. The room temperature was recommended as a better curing condition for the FA–GEO mortar incorporating the 20.0% soda residue. Although the higher porosity with CS contents may produce a lower mechanical strength of the CS–FA–GEO mortar, the obtained higher strength results show that the enhancement effect of higher chemical bonding exceeds the decreasing effect of porosity.

In this research, the 20.0% CS as a calcium additive shows the similar effect to the 20.0% soda residue on improving the mechanical strengths of 60 d. Therefore, a proper CS content can enhance the mechanical strengths of CS–FA–GEO mortars when cured at room temperature.

For the identical CS contents of 20.0%, as the pre-curing temperature increases from 25 °C to 80 °C, the flexural and compressive strengths at 60 d and 360 d decrease (Figure 9b). The 60 d flexural strength of FC20T80 reaches 1.2 MPa, which is 40.1% lower than that of FC20T25, whereas the 60 d compressive strength of FC20T80 reaches 3.5 MPa, which is 43.5% lower than that of FC20T25. Moreover, the 360 d flexural strength of FC20T80 reaches 1.4 MPa, which is 53.5% lower than that of FC20T25, whereas the 360 d compressive strength of FC20T80 reaches 4.2 MPa, which is 1.26 times lower than that of FC20T25. The increase in pre-curing temperature for nearly 2 h produces a negative effect on the mechanical strengths of CS–FA–GEO mortars incorporating 20.0% CS when cured for 60 d and 360 d. In the previous study, Bakharew [16] pointed out that the long-term pre-curing of the room temperature was conducive to the enhancement of the compressive strength of FA–GEO materials activated by the Na_2_SiO_3_ and NaOH activator solution. The results of this paper are consistent with those found by Bakharew. Therefore, T25 is recommended as a better curing condition to obtain higher long-term mechanical strengths.

The bend-press ratios can reflect the toughness of CS–FA–GEO composites to some certain degree. The addition of 20.0% CS increases the toughness, owing to the higher bend-press ratio (Figure 10a), whereas the pre-curing temperature of 80 °C decreases the toughness, due to the lower bend-press ratio (Figure 10b). In addition, for the different CS contents, the compressive strength increments of 60~360 d range from 3.3~4.0 MPa, whereas the flexural strength increments of 60~360 d range from 0.5~1.1 MPa. For the different pre-curing temperatures, the compressive strength increments of 60~360 d range from 0.6~3.3 MPa, whereas the flexural strength increments of 60~360 d range from 0.2~1.1 MPa. It can be seen that the T25 curing condition is conducive to improving the long-term mechanical properties of CS–FA–GEO mortars, which are mainly attributed to the early water loss exposed to the short-term high temperature, and, later, the worse chemical reaction without enough water [23]. Moreover, the additional 20.0% CS is more conducive to the improvement of long-term flexural strength and toughness when cured at room temperature.

A previous investigation reported that the addition of 20.0% soda residue increased the early and not later compressive strength of the FA–GEO mortar with a curing time of up to 360 d [17]. Shang, et al. [43] found that adding 20.0% GGBS increased both the early and later compressive strength of the FA–GEO mortar, which was approximately 50.0% higher than that without GGBS. Due to the different chemical components, the CS has a similar effect to GGBS, but a different effect to the soda residue on the compressive strength.

In addition, the long-term mechanical strengths increase, which mainly results from both the porosity and the chemical geopolymeric cements between particles and solutions. This may be because the free Ca^2+^ cations are firstly dissolved from CS under the high alkaline environment. Then, the dissolved Si–O tetrahedron and Al–O tetrahedron from FA produce a geopolymeric reaction in order to generate N–A–S–H gels that combine with Na^+^ cations from the alkaline activator. In addition, excessive free Ca^2+^ cations will combine with Si–O tetrahedrons or Si–O–Al tetrahedrons to produce C–S–H or C–A–S–H gels [19,43]. CS–FA–GEO composites may coexist with the same chemical reaction mechanisms as FA–GEO incorporating soda residue or slag, which produces a positive impact on long-term mechanical strengths [19,23,43]. However, the higher porosity of FA–GEO with 20.0% CS will contribute to lower mechanical strengths (see Section 3.3). Therefore, the addition of 20.0% CS is conducive to the enhancement of the long-term flexural strength, compressive strength, and toughness of the CS–FA–GEO mortar under T25, due to the main chemical binding over the physical porosity. However, the specific gel products in CS–FA–GEO composites need to be further detected using other experimental techniques.

### 3.6. Characterization of Microstructures and Gel Products in CS–FA–GEO Composites

**Figure 11:**
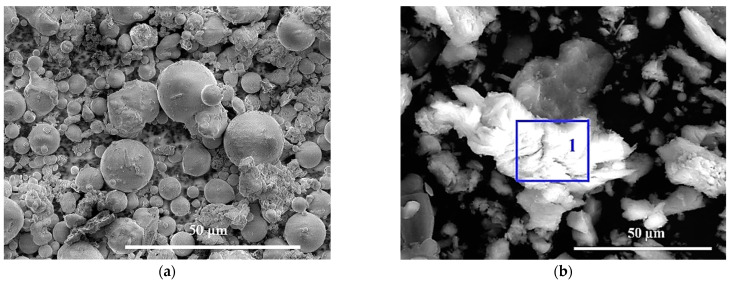

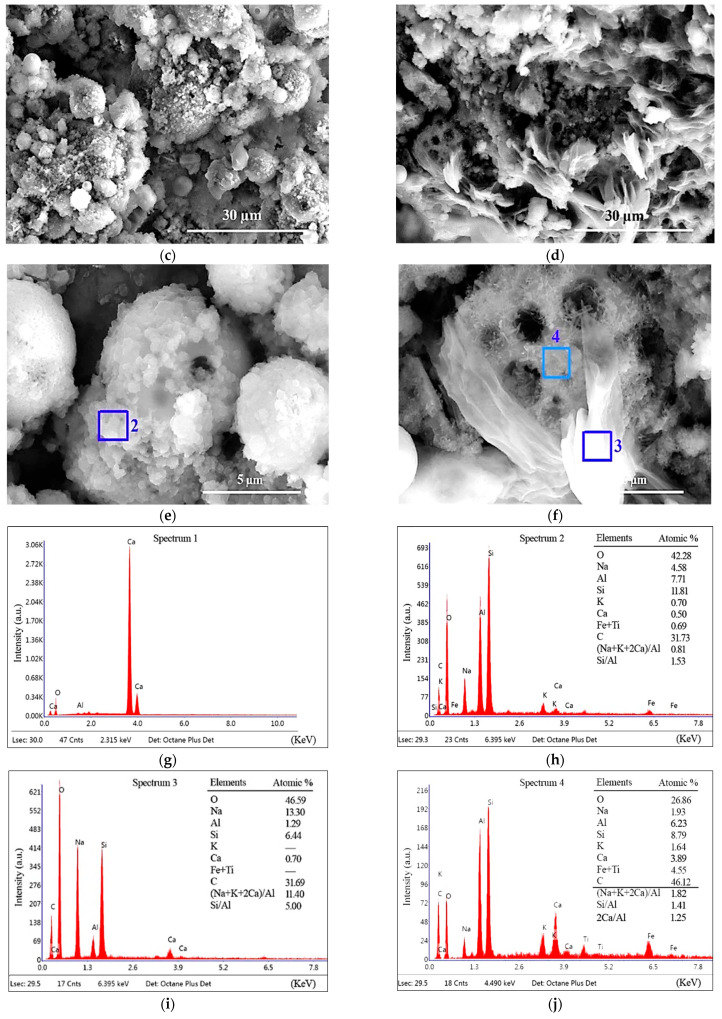
Morphologies of (**a**) FA, (**b**) CS, (**c**,**e**) FC0T25 at 360 d, and (**d**,**f**) FC20T25 at 360 d, coupled with EDS spectra of the representative points (**g**) spectrum 1, (**h**) spectrum 2, (**i**) spectrum 3, and (**j**) spectrum 4.

After the recommended pre-curing of T25 for 360 d, the microstructures and gel products were further investigated for the synthesized samples FC0T25 and FC20T25, compared to raw powders FA and CS (Figure 11a–j).

On the microscale, the solid powder FA mainly shows glassy microspheres with smooth surfaces, and consists of Si and Al elements (Figure 11a). Meanwhile, the solid powder CS presents irregular particles with corners (Figure 11b), which mainly contain Ca and O elements (Figure 11g).

Besides, the binder in FC0T25 without CS is synthesized using a FA and NaOH solution by a L/S ratio of 0.73. The main elements in the gel products include O, Na, Si, Al, C, and less K, Ca, and Fe at spectrum 2 (Figure 11e,h). Here, owing to little Ca, the high-percent element C is not derived from CaCO_3_, but from the Na_2_CO_3_ or NaHCO_3_ of surficial pan-alkali. Moreover, the main gel products are determined as N–A–S–H gels, which also contain less K, Ca, and Fe. This conclusion is consistent with the gel products in the alkaline Na_2_SiO_3_ and/or NaOH solution activated low-calcium fly-ash-based geopolymer gels mentioned in previous reports [10,19,23].

The binder in FC20T25 is prepared using the powder FA, powder CS, and NaOH solution by a L/S ratio of 0.73, which contains 20.0% CS. Compared to the FC0T25, the addition of 20.0% CS improves cementation and increases the compactness of FA–GEO composites (Figure 11c,d). From the SEM images, the microsphere of FA happens to dissolve to a greater extent, owing to the existence of CS (Figure 11e,f). Furthermore, by spectrum 3 and spectrum 4 of EDS testing, it can be seen that the substance at spectrum 3 is determined as Na_2_CO_3_ or NaHCO_3_, which is derived from the surficial pan-alkali (Figure 11f,i). The gel products at spectrum 4 are determined as the mixtures of N–A–S–H and C–S–H gels, which can be judged from the main elemental components with more O, Na, Al, Si, Ca, C, Fe, and Ti, and less Na and K (Figure 11f,j).

The change in gel products (from C–S–H gels to the coexistence of N–A–S–H and C–S–H gels) determines macro-properties and micro-structures as the additions of CS. In a previous study, the calcium additives influenced the gel products of the alkali-activated FA–GEO material (for example, backfill paste for goaf [19], geopolymer mortar [23] and engineering soil for backfill [44], etc.). According to the geopolymer theory of the alkali-metal cations (Na^+^, K^+^, and Ca^2+^) coupled with Si–O–Al and/or Si–O–Si bonds, Zhao et al. [10,19,44] proposed that the gel products of alkali-activated fly-ash-based materials were clarified and determined in the alkali-activated gels as the following Expressions (2), (3), and (4):

(Na + K + 2Ca)/Al ≥ 1.00(2)

Si/Al > 1.00 (3)

2Ca/Al ≥ 1.00 (4)

where, for the alkali-activated gels, (Na + K + 2Ca)/Al ≥ 1.00 ensured a strong enough alkali solution, including the Na^+^ or K^+^ required for alkali activation. Si/Al > 1.00 ensured that the dissolved Al in FA could totally participate in the chemical geopolymer. Further, the expression 2Ca/Al ≥ 1.00 was used to determine that the Ca^2+^ cation or calcium additive was too excessive to form the coexistence of N–A–S–H and C–S–H gels.

At spectrum 4, the above indexes in the gels were measured and calculated: (Na + K + 2Ca)/Al = 1.82 (higher than 1.00), Si/Al = 1.41 (higher than 1.00), and 2Ca/Al = 1.25 (higher than 1.00), as shown in Figure 11f,j. Therefore, the gel products of FA–GEO composites with 20.0% CS are determined as the mixtures of N–A–S–H and C–S–H gels.

## 4. Conclusions

The aim of this paper is to evaluate the effects of carbide slag (CS) on the fresh, physical, and mechanical properties of fly ash (FA)-based geopolymer (FA–GEO) composites, and to further characterize the microstructure and gel products of CS–FA–GEO composites. The feasibility was investigated regarding the application of CS as a calcium additive in the alkali-activated FA–GEO composites. The samples with different CS contents (from 0.0% to 20.0%) were manufactured and cured at different pre-curing temperatures for nearly 2 h (from 25 °C to 80 °C). The main conclusions have been obtained as follows:

(1) The additional 20.0% CS as a calcium additive reduces the fluidity and increases the conductivity of FA–GEO composites, indicating a higher ionic environment for further reaction, besides water absorption. The long-term bulk densities of 360 d are lower than those of 60 d due to the loss of water for CS–FA–GEO composites;

(2) With the increase in the CS content, the porosities of CS–FA–GEO samples increase, and the porosities also increase with pre-curing temperatures increasing from 25 °C to 80 °C for 2 h. The additional CS produces a higher chemical reaction rate and higher pore generation rate when exposed to a higher temperature. The addition of CS improves the drying shrinkage property of the FA–GEO composite. The shrinkage of FC20T25 is stable at −560 × 10^−6^ until 210 d at room temperature;

(3) Room temperature curing is recommended as the better curing condition to produce higher long-term mechanical properties of CS–FA–GEO mortars, which are attributed to the early water loss exposed to the short-term high temperature, and, later, the worse chemical reaction without enough water. Moreover, the addition of 20.0% CS is conducive to the improvement of the flexural strength, compressive strength, and toughness of CS–FA–GEO composites cured for 360 d at room temperature;

(4) The gel products in CS–FA–GEO composites incorporating 20.0% are mainly determined as the mixtures of N–A–S–H and C–S–H gels containing lower amounts of other elements, such as Fe, Ti, and K, besides that of Na_2_CO_3_ or NaHCO_3_, from the surficial pan-alkali.

In summary, the application of solid waste CS in FA–GEO composite materials is feasible. The contribution of this paper is to validate the effects of CS on the fresh, physical, and mechanical properties, as well as the microstructures and gel compositions, of CS–FA–GEO composites. It supports the wider application of CS in brick and block materials that do not require high mechanical strengths. In the next research, the higher mechanical strengths (evenly higher than 20.0 MPa) need to be further investigated for CS–FA–GEO composites at room temperature by using other useful calcium additives. It provides a better prospect for CS and FA in green and eco-friendly alkali-activated materials.

## Figures and Tables

**Table 1:** Chemical compositions of carbide slag (CS) and fly ash (FA) by X-ray fluorescence.

Compositions	CaO	SiO_2_	Al_2_O_3_	Fe_2_O_3_	MgO	Others	Loss on Ignition (1000 °C)
CS	63.13%	8.70%	0.50%	1.03%	1.20%	—	25.44%
FA	5.42%	51.10%	25.20%	7.92%	—	7.36%	3.00%

**Table 2:** Basic indexes of CS and FA.

Raw Powders	Specific Gravity	Specific Surface Area (m^2^/kg)	Amount Passing #325 Sieve	Mean Particle Size (mm)	pH ^1^
CS	1.80	420	40.00%	0.22	10.55
FA	2.45	500	73.00%	0.10	6.93

^1^ ‘pH’ refers to the pH values measured under the condition of 100% water content and 25 ± 3 °C temperature.

**Table 3:** Mixing proportions and pre-curing temperatures of CS–FA–GEO composite samples.

No.	FA (g)	CS (g)	CS Content ^1^ (%)	L/S ^2^	C/S ^3^	Na/Si Ratio	Al/Si Ratio	Ca/Si Ratio	Pre-Curing Temperature
FC0T25	450.0	0.0	0.0	0.73	1:3	0.74	0.29	0.11	T25
FC4.4T25	430.2	19.8	4.4	0.73	1:3	0.76	0.29	0.17	T25
FC8.8T25	410.4	39.6	8.8	0.73	1:3	0.79	0.29	0.24	T25
FC13.2T25	390.6	59.4	13.2	0.73	1:3	0.83	0.28	0.31	T25
FC17.6T25	370.8	79.2	17.6	0.73	1:3	0.86	0.28	0.38	T25
FC20T25	360.0	90.0	20.0	0.73	1:3	0.88	0.28	0.43	T25
FC20T40	360.0	90.0	20.0	0.73	1:3	0.88	0.28	0.43	T40
FC20T50	360.0	90.0	20.0	0.73	1:3	0.88	0.28	0.43	T50
FC20T60	360.0	90.0	20.0	0.73	1:3	0.88	0.28	0.43	T60
FC20T70	360.0	90.0	20.0	0.73	1:3	0.88	0.28	0.43	T70
FC20T80	360.0	90.0	20.0	0.73	1:3	0.88	0.28	0.43	T80

^1^ CS content refers to the mass ratios of CS to the total solid powders. ^2^ All of liquid/solid (L/S) ratios of samples are set to 0.73, which refer to the mass ratio of NaOH solution to solid powders (powder CS and powder FA). ^3^ All of cement/sand (C/S) ratios of samples are set to 1:3, which refer to the mass ratio of solid powders to standard sand.

**Table 4:** Mixing proportions of drying shrinkage samples.

No.	FA (g)	CS (g)	CS Content ^1^ (%)	L/S ^2^	C/S ^3^	Na/Si Ratio	Al/Si Ratio	Ca/Si Ratio	Curing Temperatures
FC0T25	450.0	0.0	0.0	0.73	1:2	0.74	0.29	0.11	T25
FC8.8T25	410.4	39.6	8.8	0.73	1:2	0.79	0.29	0.24	T25
FC20T25	360.0	90.0	20.0	0.73	1:2	0.88	0.28	0.43	T25

^1^ CS content, ^2^ L/S, and ^3^ C/S refer to the identical meaning in Table 3.

## Data Availability

The data presented in this study are available on request from the corresponding author. The data are not publicly available due to the privacy restrictions.
